# Glioblastoma and PARP inhibitors: the importance of patient selection to maximize therapeutic benefits. A critical review

**DOI:** 10.3389/fonc.2026.1713069

**Published:** 2026-03-31

**Authors:** Giovanni Dima, Vincenzo Di Nunno, Marta Aprile, Lidia Gatto, Alicia Tosoni, Chiara Maria Argento, Marzia Margotti, Stefania Bartolini, Enrico Franceschi

**Affiliations:** 1 Nervous System Medical Oncology Department, Istituto di Ricovero e Cura a Carattere Scientifico (IRCCS) Istituto delle Scienze Neurologiche di Bologna, Bologna, Italy; 2 Unit of Oncology, University Hospital of Pisa, Pisa, Italy; 3 Department of Translational Research and New Technologies in Medicine and Surgery, University of Pisa, Pisa, Italy

**Keywords:** blood-brain barrier (BBB) penetration, DNA damage repair, glioblastoma (GBM), molecular biomarkers, poly(ADP-ribose) polymerase inhibitors (PARPi), radiotherapy sensitization, temozolomide (TMZ), therapeutic resistance

## Abstract

Glioblastoma (GBM) is the most common malignant primary brain tumor in adult patients and has a poor prognosis despite current multimodal approaches that include surgery, radiation therapy, and chemotherapy. Local recurrence is almost inevitable, and treatment options for relapsed disease are limited. Poly-ADP-ribose polymerase inhibitors (PARPi) are standard of care in several advanced/metastatic solid malignancies including ovarian, breast, and prostate cancer. In GBM, PARPi could represent a novel promising class of therapeutic agents enhancing DNA repair defects present in specific GBM molecular subtypes. Indeed, these agents act by blocking DNA damage repair, amplifying the lethality of DNA-damaging therapies such as temozolomide (TMZ) and radiotherapy. Several PARPi have been investigated in combination with standard GBM therapy, without significant clinical improvement. However, their efficacy is strongly influenced by their ability to cross the blood-brain barrier and the presence of tumor resistance mechanisms. Clinical trials suggest that patient selection using molecular biomarkers and the timing of PARPi administration are crucial for maximizing therapeutic benefit. The choice of PARPi with optimal trapping and blood-brain barrier penetration capacity, together with molecular stratification based on genomic and expression profiles, appears crucial for the effective use of these agents in glioblastoma. This review investigates the biological mechanisms associated with PARPi within GBM tumor cells and assesses which patients could be the best candidates for PARPi treatment based on molecular profiling of the disease.

## Introduction

Glioblastoma (GBM) is the most common malignant primary brain tumor in adult patients. Overall, it is a rare malignancy with an estimated incidence of 3–4 cases per 100,000 individuals (1, 2). The prognosis for these patients remains poor, with an estimated median overall survival (OS) of 14.6 months, and a 24-month OS rate of less than 25% (3). The standard approach to therapy in the newly diagnosed setting includes surgery followed by radiotherapy with concurrent and adjuvant temozolomide (4, 5). At tumor relapse, re-surgery/re-irradiation and systemic therapies are potential options but associated with a modest clinical impact (4–6). Therefore, the research of active treatments in this setting still remains an unmet critical need.

Inhibitors of poly-ADP-ribose polymerase (PARPi) are considered promising in the treatment of glioblastoma for several reasons. Rather than exhibiting a hypermutator phenotype, glioblastoma is defined by recurrent genomic alterations affecting core regulatory networks that maintain genomic integrity, most prominently the p53, RB, and RTK/PI3K pathways. Perturbation of these signaling axes impairs DNA damage surveillance, checkpoint activation, and apoptosis, enabling continued proliferation under conditions of genotoxic stress. PARP inhibition in this setting interferes with single-strand break repair, leading to replication-associated double-strand breaks and enhanced tumor cell lethality (7).

**Figure 1:**
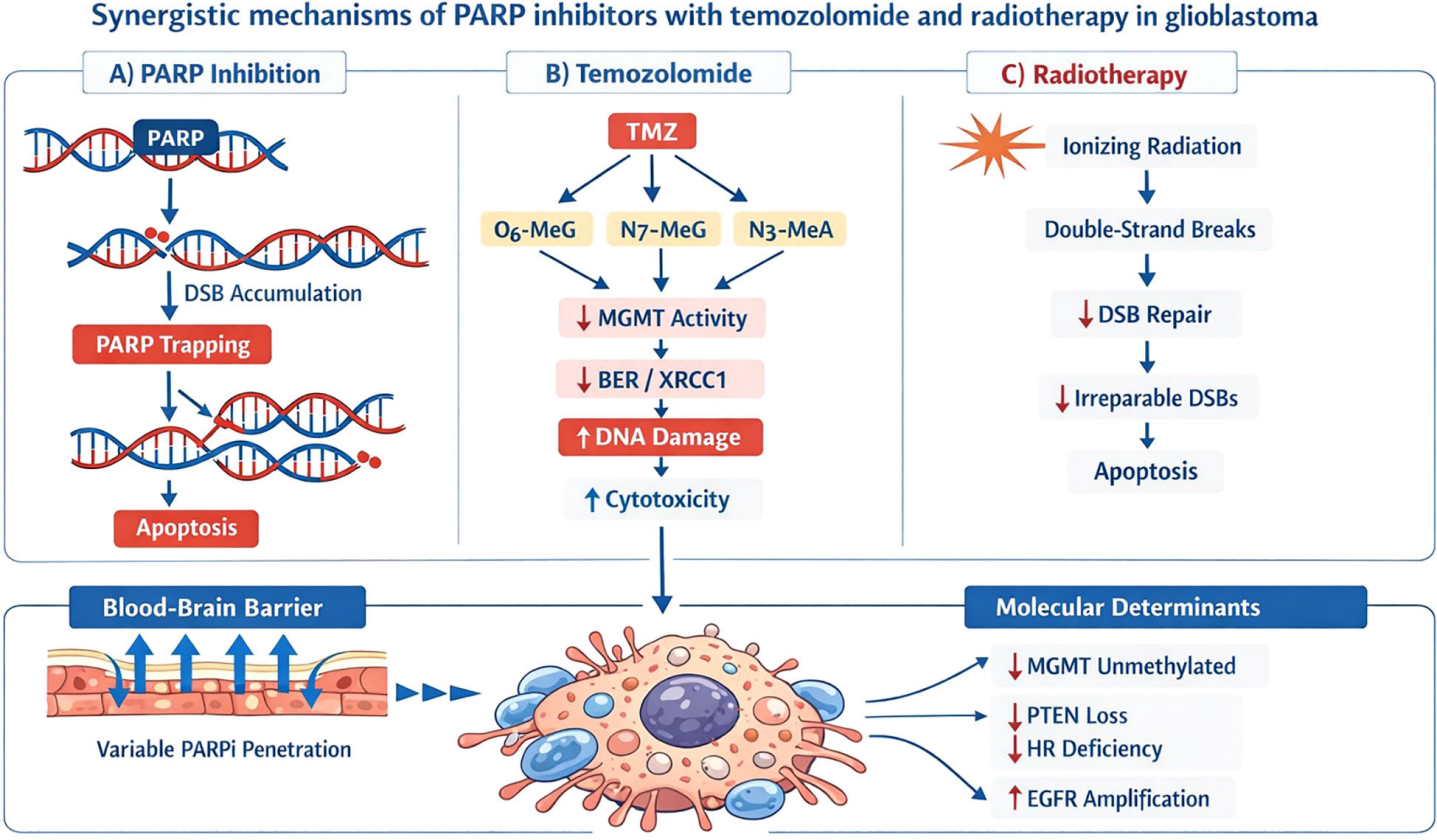
Synergistic mechanisms of PARP inhibitors with temozolomide and radiotherapy in glioblastoma.

Radiotherapy and temozolomide (standard chemotherapy for glioblastoma) cause DNA damage, increasing the formation of SSBs and DSBs. Ionizing radiation primarily induces DNA double-strand breaks, the most lethal form of DNA damage, which are repaired mainly through homologous recombination and non-homologous end joining pathways. Cells with impaired homologous recombination are therefore particularly vulnerable to radiation-induced damage, providing a biological rationale for combining radiotherapy with PARP inhibition (8). PARPi reduces the cell’s ability to repair this damage, enhancing the cytotoxic efficacy of standard therapy. The synergistic mechanisms of PARP inhibitors with temozolomide and radiotherapy in glioblastoma are summarized in **Figure 1**. This synergistic effect is particularly important in glioblastoma, where resistance to treatment is common (9). To date, available trials on PARPi in GBM showed limited clinical efficacy of these agents. Nonetheless, PARPi have very different blood-brain barrier (BBB) penetrance and trapping properties. In addition, no trials investigated these agents in a cohort of GBM with selected molecular features, including GBM associated with hereditary cancer predisposition syndromes, such as Lynch syndrome and mismatch repair deficiency (10).

This review investigates the possible clinical role of these drugs in GBM, focusing on possible strategies to enhance PARPi clinical activity and on patients more likely to benefit from these targeted agents.

## PARP Inhibitors

PARP is a ubiquitous protein modulating gene expression, cell proliferation, homeostasis, and apoptosis. The N-terminal domain is responsible for binding to DNA, the central domain called the self-modification domain regulates enzymatic activity, and the catalytic domain is located at the C-terminal end of the protein. Using NAD+ as a substrate, PARP recruits enzymes responsible for repairing genetic material by building ADP-ribose (PAR) polymers on appropriate proteins (histones, topoisomerases, etc.). Poly-ADP-ribosylation modifies the chemical-physical properties of these proteins, triggering the cascade of events that leads to DNA repair. PARPi act by blocking the enzymatic activity of PARP, thereby preventing its auto-ADP-ribosylation (auto-PARylation), and thus the dissociation of the enzyme from damaged chromatin, stabilizing the PARP-DNA complex (PARP trapping) and leading to replication fork stalling, with consequent formation of DSBs (11). PARP trapping describes the formation of stable PARP–DNA complexes when inhibitor-bound PARP1 cannot undergo auto-PARylation and release from sites of DNA damage. These trapped complexes act as physical obstacles to replication fork progression, causing fork stalling and collapse with subsequent generation of double-strand breaks. In contrast to simple catalytic inhibition, PARP trapping produces lesions that are particularly toxic to cells with impaired homologous recombination repair (12). Under physiological conditions, PARP1 functions as a rapid sensor of single-strand DNA breaks and facilitates repair through poly(ADP-ribosyl)ation-dependent recruitment of base excision repair proteins. By preventing PARylation, PARP inhibitors disrupt the assembly of these repair complexes and prolong the persistence of DNA lesions. This pharmacological effect transforms PARP from a repair enzyme into a DNA-bound obstacle, thereby amplifying the cytotoxic impact of endogenous damage and of DNA-damaging treatments (13).

PARPi present different pharmacokinetic properties affecting their potential efficacy in patients with glioblastoma. The main differences are related to trapping, half-maximal inhibitory concentration (IC_50_), and BBB penetration. The cytotoxic effects of PARPi appear to be more related to trapping activity than to direct PARP inhibition. Trapping involves PARP/PARPi binding to a broken DNA segment, creating a complex with direct cytotoxic effects, resulting in apoptosis and tumor cell death. PARPi have different strengths, resulting in different trapping activities. This could partially explain the varying clinical efficacy of PARPi (14, 15). This trapping of PARP-DNA complexes, which stabilizes the enzyme bound to damaged DNA, appears to be the most important mechanism by which PARPi lead to cell death (16). Trapping leads to the formation of PARP-DNA complexes at the site of single-strand breaks (SSBs), hindering DNA repair and promoting the conversion of SSBs into lethal double-strand breaks (DSBs). This is the main mechanism of cytotoxicity and cell death (17).

This model is supported by the fact that PARP inhibitors show significant differences in cytotoxic potency despite having similar IC_50_ values for the catalytic inhibition of PARP—that is, the concentration of a drug needed to inhibit a biological process or enzyme activity by 50%. The IC_50_ measures the potency of an inhibitor, with lower values indicating higher potency. This suggests that the trapping capacity contributes significantly to their antitumor efficacy. Indeed, talazoparib is the PARPi associated with the strongest trapping activity. This PARPi traps PARP to DNA with ~100-fold greater potency compared to olaparib and rucaparib and nearly ~1,000-fold compared to veliparib. Although PARP trapping is thought to be the main mechanism behind cytotoxicity, a combination of both PARP catalytic inhibition and trapping is important to the mechanisms of action of PARP inhibitors (18). All four approved PARP inhibitors (Niraparib, Olaparib, Talazoparib, and Veliparib) are selective, potent inhibitors of PARP enzymes approved for multiple HRD (homologous recombination deficiency) cancers. The IC_50_ in wild-type cells for inhibiting PARP catalytic activity is 4 nM for talazoparib, compared to 6 nM for olaparib, 21 nM for rucaparib, and 60 nM for niraparib (18). However, as mentioned previously, PARP inhibitor cytotoxicity is better predicted by trapping ability, with talazoparib potency ~100- to 1000-fold higher than other approved PARP inhibitors (15). Both talazoparib and niraparib have longer half-lives of 90 h and 36 h, respectively, compared to the half-lives of olaparib and rucaparib of 15 h and 26 h, respectively.

The inhibitors differed markedly in their ability to trap PARP, with Talazoparib showing the highest potency, followed in descending order by Niraparib, Olaparib, Rucaparib, and Veliparib. This ranking highlights the substantial differences in efficacy among these compounds (15). Using a PARPi that has a stronger trapping mechanism than the others might be a good strategy. Although talazoparib exhibits the highest PARP-trapping potency among clinically available inhibitors, this property alone does not guarantee therapeutic efficacy in glioblastoma. DNA damage response networks are highly interconnected, and tumor cells can activate compensatory pathways that mitigate the effects of PARP inhibition. In particular, PARP inhibition induces replication stress that increases cellular reliance on ATR/CHK1 signaling to stabilize stalled replication forks and prevent catastrophic DNA damage, thereby enabling survival despite extensive genomic injury. Alternative end-joining mechanisms mediated by polymerase theta (POLQ) and replication fork protection processes may further contribute to resistance. These adaptive responses, together with tumor heterogeneity and pharmacokinetic constraints, likely underlie the limited clinical activity observed with highly potent PARP inhibitors in GBM (19).

Finally, the ability to cross the BBB is another important issue to consider.

The permeability of PARP inhibitors across the BBB depends on multiple factors, including pharmacokinetic and molecular chemical-physical properties, such as lipophilicity, molecular weight, and substrate for efflux transporters such as P-gp (P-glycoprotein) and BCRP (Breast Cancer Resistance Protein) (20, 21). (**Table 1**). These transporters function as efflux pumps that actively expel drugs and xenobiotics from cells, reducing their intracellular concentrations and contributing to drug resistance. Their expression at the BBB plays a critical role in regulating substance passage, thereby protecting the brain from potentially harmful compounds (20).

**Table 1:** Concentration in brain tissue of PARP inhibitors.

PARP inhibitor	Brain tissue/plasma ratio	Note
Olaparib (22)	< 0.1	Olaparib is a substrate for P-gp and BCRP efflux transporters, which strongly limit its permeability through the BBB. Olaparib shows limited efficacy against brain metastases in preclinical models
Niraparib (23)	0.3-0.5	Niraparib has greater lipophilicity and lower affinity for efflux transporters than olaparib. This suggests a potential improvement in efficacy against brain tumors or metastases.
Talazoparib (24)	0.1-0.2	Despite its potency, its penetration into the brain is moderate probably due to recognition by P-gp.
Rucaparib (25)	0.1-0.15	Rucaparib crosses the BBB poorly because it is a substrate of the efflux transporters P-gp and BCRP, which limit its concentration in the brain.
Veliparib (26)	0.47	In mouse models of glioblastoma, veliparib shows more favorable intracranial distribution than other PARP inhibitors In a phase II clinical trial in patients with brain metastases from NSCLC, veliparib demonstrated penetration into the central nervous system

Importantly, BBB penetration alone does not ensure adequate drug exposure within tumor tissue or therapeutic activity. Differences in efflux transport, tumor heterogeneity, and binding to intracellular targets can result in intratumoral concentrations that diverge from bulk brain measurements. The clinical failure of veliparib, despite relatively favorable BBB penetration, underscores this issue and suggests that trapping potency and pharmacodynamic effects may be more critical determinants of efficacy than drug distribution alone.

## PARP inhibitors in GBM

Preclinical studies suggested that PARPi could be clinically active in GBM. Temozolomide is an alkylating agent with proven clinical efficacy on glioblastoma. It induces DNA methylation predominantly at the N7 position of guanine and the N3 position of adenine, lesions that are primarily repaired through the base excision repair (BER) pathway, while O6-methylguanine lesions trigger mismatch repair (MMR) processing that can ultimately lead to cytotoxic DNA damage. The effectiveness of TMZ therefore depends largely on the capacity of tumor cells to engage BER and MMR pathways to resolve these lesions (27). This agent mediates methylation on the guanine residue O6 (O6-meG), residue N7 (N7-meG), and adenine residue 3 (N3-meA) (28). Glioblastoma cells mediate resistance by eliminating O6-meG through PARylation (PARP-mediated) of *MGMT* or by activating microsatellite mismatch repair (MMR). Base excision repair (BER) is another PARP-mediated mechanism employing PARP and X-ray cross-complementing protein 1 (*XRCC1*) by which GBM cells eliminate N3-meA and N7-meG (28). Therefore, PARP inhibition leads to the accumulation of O6-meG by preventing *MGMT* PARylation, and it also impairs the repair of N3-meA and N7-meG via the base excision repair (BER) pathway. Both these actions lead to DNA damage and cell apoptosis (28).

PARP interacts with and PARylates *MGMT* to remove damaged DNA, independent of BER, in response to TMZ treatment. In GBM, *MGMT* status is a known predictive and prognostic factor. Methylation of *MGMT* leads to *MGMT* inactivation, thus improving the efficacy of alkylating agents and radiation therapy. GBM cells with unmethylated *MGMT* can easily develop resistance to alkylating agents through PARP/*MGMT*-mediated O6-meG elimination. Preclinical data suggest that tumors with active *MGMT* expression may be more sensitive to PARP inhibition in combination with temozolomide, as PARPi can interfere with *MGMT*-dependent repair mechanisms. However, current clinical evidence does not yet provide clear confirmation of this hypothesis. Notably, randomized trials such as VERTU did not demonstrate a significant clinical benefit of PARPi-based strategies in *MGMT*-unmethylated glioblastoma. These findings highlight the translational gap between preclinical rationale and clinical outcomes and underscore the need for improved patient selection strategies (19). These same mechanisms also mediate sensitivity to radiation therapy. In 2008 a study by Dungey et al. (29) and in 2011 a study by van Vuurden et al. (30) showed that olaparib improves radiosensitivity in glioma cell lines. PARPi are clinical drugs that target altered DNA damage response in cancer. The mechanism of action of PARPi is believed to be the physical trapping of PARP onto damaged DNA, yet how PARP “traps” has remained elusive. Against this background, the best setting for testing this drug is the adjuvant phase of treatment in which both TMZ and radiation therapy are administered.

PARP inhibitors are most effective in tumors with defects in homologous recombination repair, typically associated with BRCA1/2 mutations. However, classical BRCA mutations are uncommon in glioblastoma, particularly BRCA2 alterations. Nevertheless, a subset of GBM may exhibit a “BRCAness” phenotype due to noncanonical mechanisms of homologous recombination deficiency, including epigenetic silencing of HR-related genes, alterations in DNA repair pathways, or functional impairment of BRCA1-associated networks (31). In this context, HRD scoring systems developed for other malignancies have not been systematically validated in glioblastoma, and their clinical utility for patient selection remains uncertain. Preclinical studies suggest that PARP inhibition may still be effective in this setting, as GBM models with BRCA mutations or PTEN deficiency show enhanced sensitivity to PARPi, particularly in combination with temozolomide (32, 33). In GBM, PARPi showed potential clinical effect with a favorable safety profile (34). The most common side effects are dose-related, mostly reversible decreases in lymphocytes and WBC, moderate diffuse germ cell depletion affecting mainly spermatogonia and spermatocytes, a possible bronchodilator effect, inhibition of the IKr channel, no effects on QT, nausea, fatigue, and decreased appetite (9).

The promising activity in terms of response and acceptable tolerability and safety profile led to planning dedicated clinical trials in glioblastoma. In 2021, the VERTU study, a Phase II trial, evaluated the preliminary efficacy and safety of veliparib in patients with glioblastoma and unmethylated *MGMT*. 125 participants were randomized 2:1 to veliparib and radiotherapy, followed by adjuvant veliparib and temozolomide versus standard care. The primary objective was progression-free survival rate at 6 months (PFS-6) in the experimental arm. PFS-6 was not significantly improved with the addition of veliparib, resulting in 46% versus 31% (Hazard ratio: 0.78; 95% CI: 0.54–1.15) (34).

In 2024, Sarkaria et al. conducted a Phase II/III clinical study in 447 patients with newly diagnosed glioblastoma, randomized 1:1 to veliparib + temozolomide versus placebo + temozolomide (35). The primary endpoint for Phase II was PFS, and for Phase III was OS. The addition of veliparib to temozolomide did not improve OS, which was similar between the two arms, with a median of approximately 25 months versus 28 months and a hazard ratio close to 1. A modest difference was observed in the rate of long-term survivors favoring veliparib over 24–42 months of follow-up. Despite the negative results, the observed positive OS trend suggested that a subset of GBM might be uniquely sensitive to the combination. It is possible that some patients present molecular defects in key DNA repair pathways, making them more likely to benefit from inhibition of parallel, PARP-dependent repair pathways combined with temozolomide-induced DNA damage (35). In 2025, Stefan et al. presented results of a Phase I study on the safety of olaparib in patients diagnosed with glioblastoma concomitant with the Stupp Protocol. Median PFS and OS were 6.2 and 19.8 months, respectively. The safety profile was acceptable, and clinical outcomes were promising (4). Although PARPi are associated with a favorable toxicity profile, their combination with temozolomide increases the risk of severe hematological toxicity. Novel PARPi with improved toxicity profiles, higher trapping activity, and better BBB penetration are under investigation. Among these, NM-S-03305293 is a PARP-1 inhibitor that can deliver selective PARP-1 trapping while sparing PARP-2, resulting in a favorable toxicity profile and limited hematological toxicity compared to other PARPi. This agent also appears to cross the BBB, achieving high brain concentrations. A multicenter, open-label, single-arm Phase I/II study is ongoing on NM-S-03305293 combined with temozolomide in adult patients with diffuse gliomas (Phase I) and IDH wild-type glioblastoma (Phase II) at first relapse (NCT04910022). Pamiparib is another PARPi with favorable hematological toxicity, high BBB penetration, and strong trapping activity. A phase I trial is assessing Pamiparib plus temozolomide in adolescents and young adults with *IDH1/2*-mutant grade I–IV glioma, newly diagnosed or recurrent (NCT03749187).

An overview of ongoing clinical trials is available in **Table 2**.

**Table 2:** Clinical trial involving PARP inhibitors in glioblastoma.

Study name	Phase	Sample size	Design
Vertu Study (34)	II	125	Veliparib + RT → Veliparib + TMZ TMZ + RT → TMZ
NCT02152982	II/III	447	Veliparib + RT → Veliparib + TMZ TMZ + RT → TMZ
NCT03581292	II	74	Veliparib + RT + TMZ → Veliparib + TMZ TMZ + RT → TMZ
Blakeley et al. (36)	II	81	RT + TMZ + Iniparib
NCT04221503	II	30	Ttf + Niraparib
Fanucci et al. (37)	II	15	Olaparib
TAC-GreD	II	33	RT + CBDCA + Talazoparib
NCT02974621	II	70	Cediranib + Olaparib Beva
NCT05076515	II	20	Niraparib + RT
NCT03991832	II	29	Olaparib + Durvalumab
NCT03561870	II	35	Olaparib
NCT06388733	II	450	Niraparib + RT → Niraparib TMZ + RT → TMZ
Baxter et al. (38)	I/II	52	Veliparib + RT → TMZ + Veliparib
NCT04910022 ongoing	I/II	150 estimated	NMS-03305293 + TMZ
NCT03749187 ongoing	I	78 estimated	Pamiparib + TMZ

Given the good predictions in preclinical studies, the failure of clinical trials combining PARPi with standard of care could be explained by our current inability to select the right type of population with the right molecular characteristics.

## Mechanisms of resistance to PARPi

The lack of efficacy of PARPi could be explained by the existence of innate and/or acquired mechanisms of resistance to these drugs. As previously argued, *MGMT* methylation status may influence response to PARP inhibition, although its predictive value remains uncertain in clinical settings. PARPi increase TMZ sensitivity and selectivity in *MGMT*-unmethylated GBM by regulating *MGMT* activity through a mechanism independent of BER (19). *MGMT* expression provides resistance to TMZ in a PARP-dependent manner, and PARPi do not sensitize cells to TMZ when *MGMT* activity is inhibited, suggesting that *MGMT* is required for this function (39).

Other than *MGMT*, additional genes could drive innate or acquired resistance to PARPi. In tumors with Phosphatase and Tensin homolog (*PTEN*) deficiency, the combination of PARPi had synergistic cytotoxic effects (40). In GBM, *PTEN* deficiency occurs in about 40% of cases (7). *PTEN* has nuclear functions, including transcriptional regulation of the *RAD51* gene, whose product is essential for homologous recombination (HR) repair of DNA breaks. Loss of *PTEN* in astrocytes results in increased DNA double-strand breaks that are poorly repaired due to compromised HR. This opens the possibility that patients with *PTEN*-null GBMs may be treated with PARPi (41). *EGFR* amplification occurs in 40–55% of GBM (7) and is associated with increased reactive oxygen species (ROS) and subsequent upregulation of DNA-repair pathways to counter elevated oxidative stress, rendering the tumor more vulnerable to PARPi (19). Dysregulation of the p53 pathway is a hallmark of glioblastoma. TP53 mutations are observed in a substantial proportion of tumors, and PTEN loss, frequent in GBM, can further attenuate p53 signaling through multiple mechanisms. This combined disruption undermines DNA damage checkpoints and apoptotic responses, allowing proliferation despite genomic stress (7). There is no evidence that p53 mutations directly confer PARPi resistance, but p53 disruption affects how glioblastoma cells process DNA damage mediated by topoisomerase I inhibitors (42). DNA damage triggered by TOP1 inhibitors can be repaired via PARP and HR with *RAD51*, so theoretically, p53 mutation could predispose cells to increased PARP activity. The main biomarkers and molecular signatures associated with PARP inhibitor sensitivity in glioblastoma are summarized in **Table 3**.

**Table 3:** Biomarkers and molecular signatures associated with PARP inhibitor sensitivity in glioblastoma.

Biomarker/Signature	Mechanism	Expected effect on PARPi response
*MGMT* unmethylated	Highly active *MGMT* depends on PARP for O6-meG repair	↑ Sensitivity to PARPi + TMZ
*BRCA1/2* mutations/HRD phenotype	Defective HR → PARPi synthetic lethality	↑ Sensitivity
*PTEN* loss	Reduced *RAD51* expression → HR impairment	↑ Sensitivity
*EGFR* amplification	↑ ROS → ↑ reliance on PARP-mediated repair	↑ Sensitivity (especially to talazoparib)
MMR deficiency (Lynch syndrome)	Elevated mutation load → ↑ reliance on PARP-mediated repair	Possible ↑ sensitivity
*IDH1/2* mutations	Epigenetic reprogramming → impaired DNA repair	Context-dependent sensitivity

## Conclusions

The study of PARPi in glioblastoma represents a promising and necessary area of neuro-oncology research. It is extremely important to adequately select patients, request the correct molecular profiling, evaluate resistance factors, assess prognostic factors, and choose from a class of drugs the one that has the most suitable characteristics for the tumor microenvironment.
